# Microbial Degradation of Rubber: Actinobacteria

**DOI:** 10.3390/polym13121989

**Published:** 2021-06-17

**Authors:** Ann Anni Basik, Jean-Jacques Sanglier, Chia Tiong Yeo, Kumar Sudesh

**Affiliations:** 1 Ecobiomaterial Research Laboratory, School of Biological Sciences, Universiti Sains Malaysia, Penang 11800, Malaysia; annbasik@gmail.com; 2 Sarawak Biodiversity Centre, Km. 20 Jalan Borneo Heights, Semengoh, Kuching, Sarawak 93250, Malaysia; jjsanglier.esperanza@gmail.com (J.-J.S.); cyeo@sbc.my (C.T.Y.)

**Keywords:** latex clearing protein, rubber, degradation, actinobacteria, distribution, diversity

## Abstract

Rubber is an essential part of our daily lives with thousands of rubber-based products being made and used. Natural rubber undergoes chemical processes and structural modifications, while synthetic rubber, mainly synthetized from petroleum by-products are difficult to degrade safely and sustainably. The most prominent group of biological rubber degraders are Actinobacteria. Rubber degrading Actinobacteria contain rubber degrading genes or rubber oxygenase known as latex clearing protein (*lcp*). Rubber is a polymer consisting of isoprene, each containing one double bond. The degradation of rubber first takes place when *lcp* enzyme cleaves the isoprene double bond, breaking them down into the sole carbon and energy source to be utilized by the bacteria. Actinobacteria grow in diverse environments, and *lcp* gene containing strains have been detected from various sources including soil, water, human, animal, and plant samples. This review entails the occurrence, physiology, biochemistry, and molecular characteristics of Actinobacteria with respect to its rubber degrading ability, and discusses possible technological applications based on the activity of Actinobacteria for treating rubber waste in a more environmentally responsible manner.

## 1. Rubber—Polyisoprenes

### 1.1. Natural Rubber (NR)

**Figure 1:**
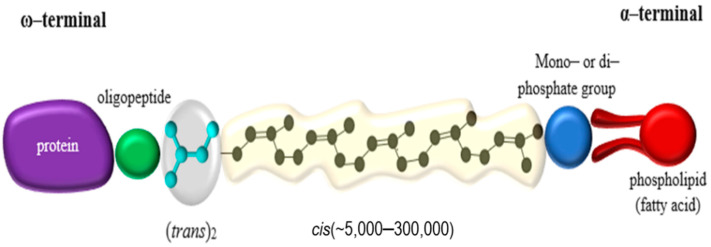
NR Polymer Chain.

A large fraction of the organic biomass on earth consists of biopolymers; such as polysaccharides, polyamino acids (proteins), polyconiferylalcohols (lignins), polyhydroxyalkanoic acids (PHAs), and polyisoprenes (rubbers) [1]. Natural rubbers (NR) farmed from *Hevea brasiliensis* Muell. Arg—comprising 99% of the world market—and *Parthenium argentatum* (guayule rubber) are produced commercially [2]. NR has a (*cis*)-1,4-polyisoprene as its polymer backbone. The backbone consists of isoprene units (C_5_H_8_) each containing one double bond in the *cis* or *trans* configuration, while 3 *trans*-isoprene units are found at one end of the molecule followed by several hundred to a few thousand *cis*-isoprene units (Figure 1) [3]. Most rubber-accumulating plants—and there are more than 2000 dicotyledons, and some fungi known to do so [4]—synthesize the polymer with the isoprene units in the *cis*-configuration [5]. Some species, such as *Manilkara chicle* or *Palaquium gutta*, however, synthesize the *trans*-polymer producing rubbers known as chicle or gutta-percha [6].

The NR latex of *H*. *brasiliensis* origin is composed of 25–35% (*w*/*w*) polyisoprene; 1.0–1.8% (*w*/*w*) protein; 1–2% (*w*/*w*) carbohydrates; 0.4–1.1% (*w*/*w*) neutral lipids; 0.5–0.6% (*w*/*w*) polar lipids; 0.4–0.6% (*w*/*w*) inorganic components; 0.4% (*w*/*w*) amino acids, amides, etc.; and 50–70% (*w*/*w*) water (Figure 1) [3]. The polymer is present in 3- to 5-µm so-called rubber particles, which are covered by a layer of proteins and lipids [7]. By virtue of its molecular structure, NR latex is a crossed-linked polymeric material that is highly flexible and extensible.

### 1.2. Production and Usage

NR is an essential raw material that is used to manufacture up to 50,000 (99% commercially used NR) different rubber and latex products [8,9]. The discovery of NR is attributed to the Olmec (also known as the “rubber people”) one of the first major civilizations in what is known as the Gulf of Mexico today, around 1600 B.C [10]. However, the first practical application in the rubber industry was discovered in 1839 when Charles Goodyear accidentally dropped rubber and sulfur on a hot stovetop and discovered vulcanization, a chemical transformation that improves NR’s elastic properties [11]. At the end of the 1800s, the automobile industry and the resulting need for tyres drove an upsurge of the then nascent rubber industry [12]. Prior to conversion into rubber products, latex from the rubber tree undergoes several manufacturing processes whereby chemicals are added to act as preservatives, anticoagulants, vulcanizing agents, and antioxidants [13].

### 1.3. Synthetic Rubber (IR)

In 1909, synthetic rubber or isoprene rubber (IR) was prepared by Fritz Hofmann [14], but due to their different properties, the usage of both NR and IR continued to increase 2.6-fold and 1.6-fold respectively from 1990 to 2017 [15]. IR refers to an artificial elastomer, mainly synthesized from by-products of the petroleum refining process [16]. There are approximately 20 different chemical types of IR, with different grades of rubber in each of those chemical categories [17]. Many of the IR consist of a mixture of copolymers whereby, specific properties such as high temperature resistance, good resistant to abrasion, strength, etc., are achieved by changing the composition of the copolymers. Both NR and IR differ in micro-structure, but both have isoprene as the main chain.

### 1.4. Rubber Wastes—Mitigation and Drawbacks

Knowledge on the fate of rubber materials in nature is still limited. Rate of decomposition depends on the type of rubber, its composition, and the environment; rubber bands take up to a year, latex glove take several months to years, rubber boot soles (synthetic rubber) take 50–80 years and tyre takes up to 2000 years to decompose [18]. Sustainability efforts for managing waste rubber products must include self-remediation through biodegradation of rubber (pre-consumer and post-consumer) in waste sites [19].

Approximately 60% of the worldwide consumption of rubber is attributed to the global tyre manufacturing industry, while the remaining rubber consumption is used to manufacture a wide variety of products such as rubber boots, rubber mulch, rubber bands, and more [17]. Tyre rubbers usually consist of 40–50% rubber (styrene-butadiene rubber, NR, and butyl rubber), 25–40% carbon black, and 10–15% low-molecular-weight additives [20]. There is currently no sustainable and environmentally friendly method of properly recycling used car tyres into new ones, or for converting the rubber polymer into other industrially useful organic compounds [21].

Landfill and stockpiles: Each year, 1.5 billion end-of-life tyres (ELTs) enter the environment globally, the majority are held in stockpiles or buried in landfills, while some are converted into by-products such as fuels, leaving only a small fraction which are recycled [22,23,24]. Burying rubber products in municipal solid waste landfills avoids processing costs but does nothing to ease the disposal problems associated with rubber waste products [25]. In 2003, an EU directive made the dumping of tyres in landfill sites more expensive and difficult, leading to a huge increase in illegal dumping of tyres all over the UK and Europe [21]. The disposal of tyres is becoming less favorable due to lack of space and occasional underground fires which cause great damage to the local environment through air, soil, and groundwater pollution [26]. Rubber materials are not inherently biodegradable within landfills. This leaves the material stagnant for hundreds of years [19] and creates ideal breeding grounds for mosquitoes and other disease vectors causing deadly outbreaks of malaria, dengue fever and encephalitis [26]. Discarded materials in the landfill also leach small molecular weight additives that can be toxic. Exposed tyres are also susceptible to sporadic fires that releases mutagenic fumes and ash material into the immediate environment [23,27].

Pyrolysis: Pyrolysis or thermal degradation (up to 800 °C) produces tyre-derived fuel known as tar pyrolysis oil (TPO) that has similar properties to diesel fuel. TPO can be used as heating, power generation, and automobile fuels, or in biorefineries [28]. The thermal degradation of tyres, however, requires a complex setup, high capital cost, skilled operators, and supplemental fuel to initiate and maintain the incineration process [29]. Mismanagement of the thermal degradation process can cause air pollution (release of greenhouse gases like CO, NO*_x_*, and SO_2_) and water pollution from runoff oil [30].

Recycling and rethreading: Unused tyres converted into tyre crumbs can be repurposed into road asphalt, playground rubber floor, athletics or cycling tracks, and modifying the structural properties of concrete. Used tyres can also be rethreaded; in the process, the degraded thread of a used tyre in good condition can be ground away, and a new thread rubber strip fused to the old carcass by vulcanization [31]. Although with rethreading almost 90% of the tyre is preserved, only tyres that meet industrial standards can be used. Safety issues and regulations governing rethreaded vehicle tyres also limit the market for rethreaded tyres and make it a less-profitable market. Moreover, rethreading still does not fully address the environmental impact of used rubber products as leaching and particle run-off still occur.

Direct use: Discarded tyres are also directly converted into port and ship fenders, slope protection levees, road traffic wall screens, signposts, marine aquaculture reefs and used in amusement facilities as bumpers [32]. However, these uses are limited. The attempt to use tyres to build artificial reefs unfortunately failed when it was found that marine life could not attach to the surface. The tyres themselves also collided with nearby natural reefs during storms damaging them.

Microplastics: Tyre abrasion resulting in miniaturized wastes (1 µm to 5 mm in diameter), has been reported to be among the main source of microplastics pollution in the environment [33]. These microplastics are mainly washed into the oceans. Due to their large area to volume ratio, they’d adsorb hydrophobic pollutants and toxins from the aquatic system (e.g., DDT, PCBs, flame retardants) before entering the food chain and being concentrated up the marine food chain [34,35]. In Switzerland, it is estimated that since 1988, 218 ktons of rubber particles have been deposited in road-side soils (74%), surface water (22%) and in soils (4%) [36]. In San Francisco Bay each year, rainfall washes more than 7 trillion microplastics left behind on streets into the water system, an amount 300 times greater than the microfibers that are shed from polyester materials and microplastic beads used in beauty products [37]. Studies show that fish have absorbed some persistent organic pollutants from plastics and subsequently developed lesions on their livers, which are precursors to cancer [38]. Plastic has also been discovered in the faeces of people, presumably from ingestion of plastic containing food, who took part in a worldwide study, but we are yet understand the impact of these micropollutants to mankind [39]. Many countries are confronting the problems posed by rubber wastes and are seeking to identify useful economic and regulatory techniques for managing them [40]. One creative idea is the use of so-called rain gardens, designed to remove well-known toxins and metals, capturing more than 90% of the microplastics [37].

Whether the rubber products are reused, recycled, or discarded, their end products still enter the environment mainly as microparticles and are very slowly degraded. It is important to understand the rubber biodegradation mechanism and discover the microorganisms involved to address the increased number and amount of rubber products being used to date.

## 2. Biodegradation: Roles of Microbes

The biodegradation of wastes is gaining interest worldwide with an emphasis on producing more easily degradable products or to improve the degradation process for existing products. Biodegradation is an environmentally friendly alternative to conventional disposal methods, wherein complex organic compounds in commercial products are biologically broken down by microorganisms into cell biomass and less complex compounds, and ultimately to water, and either carbon dioxide (aerobically) or methane (anaerobically) [19]. For microorganisms to utilize polymers as a nutrient source, an initial oxidation of polymers through abiotic process, such as exposure to ultraviolet (UV) irradiation, heat and/or chemicals in the environment is required [40]. However, some microorganisms including rubber degraders can initiate the oxidation process on their own (biodeterioration), whereby secreted enzymes (rubber oxygenase) cause biofragmentation of the polymers followed by bioassimilation of small cleavage fragments (molar mass less than 500 g mol^−1^) [41,42].

Taysum (1966) observed that NR did not accumulate in the soil of a 35-year-old plantation of rubber trees in Malaysia and speculated that there were agents present in the soil which degraded the rubber [43].

### 2.1. Rubber Degraders: Who, Where, How?

Rubber degrading strains of bacteria were mainly isolated from samples collected at rubber dense areas such as rubber plantations (e.g., soil, aged latex), from disintegrated rubber samples and rubber waste sites (e.g., water treatment pond). The collected samples, such as soil, are further enriched by adding rubber materials (e.g., latex glove) and left to incubate prior to isolation. Isolation of the strains are then carried out by plating dilutions of enriched culture on NR latex overlay agar plates. However, not all strains that can grow on NR latex agar contain rubber oxygenase, therefore it is common to amplify rubber degrading genes through PCR for confirmation.

Pure microbial isolates can also be directly transferred or streaked onto NR latex agar to screen for clear zone formation (see Section 2.1.1). 

Generally, rubber degrading strains can be divided into two types—those that produce clear zones on latex agar media and those that are adhesive contact strains [44]. In 1985, the microbial degradation of NR was first published, using *Nocardia* sp. strain 835A to degrade NR vulcanizates [45]. Since then, more than 100 rubber-degrading bacteria have been identified from different habitats, most belonging to the phylum, Actinobacteria (*Streptomyces*, *Gordonia*, *Nocardia*, *Rhodococcus*, and *Actinoplanes*), rarely found among Gram-negative bacteria, Myxobacteria and fungi [46,47] (details in Section 3.2, Section 3.4, and Section 6.4). Rubber degrading bacteria converts rubber into derivatives such as isoprenoids, aldehydes, keto groups and 12-oxo-4,8-dimethyltrideca-4,8-diene-1-al (ODTD) that can be used as value-added molecules, biofuels, polyurethanes, or other polymers [48].

### 2.1.1. Clear Zone Formers

**Figure 2:**
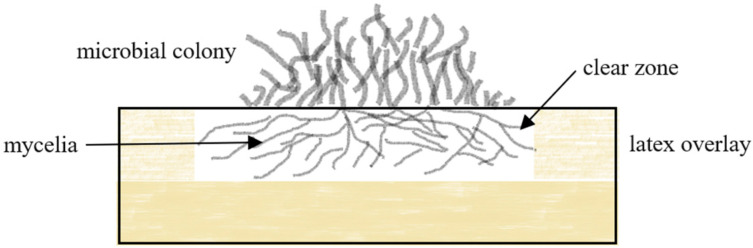
Growth of clear zone formers on NR latex overlay agar. Microbial mycelia penetrate latex agar and releases latex clearing protein enzyme cleaving the polymers double bond.

Clear zones on NR latex agar can be caused by both rubber oxygenase (*rox*) and latex clearing protein (*lcp*). These enzymes are secreted extracellularly into the medium by the strain’s mycelial corridors, diffuse into the agar and cleave the polymer to mixtures of low molecular products (C_20_, C_25_, C_30_) and higher oligo-isoprenoids as end products that are taken up by the bacteria [48]. The degradation of the NR latex layer on the agar plate subsequently leads to the formation of translucent halos [49], hence the name, clear zone formers (Figure 2). Latex overlay agar was prepared by the overlay technique, whereby, a bottom layer of mineral salts agar was allowed to solidify in a petri dish, and then was overlaid with the same agar supplemented with 0.2% (*v*/*v*) purified latex. This technique was used to isolate rubber degrading fungi and bacteria, such as *Cladosporium*, *Fusarium*, *Paecilomyes*, *Phoma*, *Actinoplanes*, *Streptomyces*, and *Micromonospora* [50].

### 2.1.2. Adhesive Contact Strains

**Figure 3:**
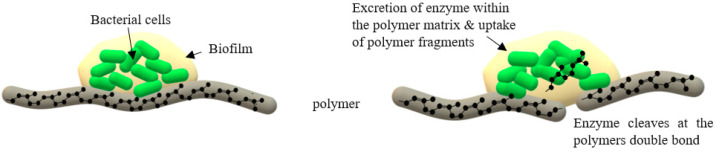
Microbial producing biofilm for attachment and enzyme excretion onto the polymer surface.

Members of the second group do not produce translucent halos but require direct contact with the rubber substrates to exert their biodegradation effects [51]. These strains consist of mycolic acid producing strains including coryneform (*Corynebacteria* and *Rhodococcus*) and nocardiaform (*Gordonia*, *Mycobacterium*, and *Nocardia*) that form biofilms which enable adhesive bacterial growth. Biofilm formation is a general microbial strategy for survival as well as for utilizing solid substrates for growth, especially in low-nutrient environment [52]. Mycolic acids are important for the formation of biofilms (Figure 3). The production of surface-active compounds (SACs) has been reported for several *Gordonia* strains which is important in degradation of water insoluble and hydrophobic pollutants [53]. The surfactants are also thought to be important for the formation of biofilms and enabling the strains to get into direct contact with the solid and water insoluble polymer during adhesive growth [54].

## 3. Rubber Degrading Enzymes: Rubber Oxygenase

Rubber degrading enzymes, also known as rubber oxygenase, are found in both Gram-positive and Gram-negative bacteria. Three rubber oxygenases have been discovered to date, namely latex clearing protein (*lcp*), rubber oxygenase A (*rox*A), and rubber oxygenase B (*rox*B) (Table 1). Despite their inherent structural differences, all three enzymes are dioxygenases that catalyze oxidative cleavage of the *cis* double bonds in the polymer backbone, generating carbonyl and aldehyde end groups in the process.

### 3.1. Latex Clearing Protein (lcp)

Rubber degrading Actinobacteria produces latex clearing protein (EC 1.13.11.87) and the functionality of *lcp* gene and its enzyme product were confirmed by several studies made though gene deletion, cloning, protein expression and functional studies [7,8,57].

A study conducted demonstrated *Streptomyces* sp. K30 (*Lcp*K30) mutant (*lcp* gene deleted) was unable to degrade poly(*cis*-1,4-isoprene), while heterologous expression of *Lcp*K30 in non-rubber degrading strains (*Streptomyces lividans* TK23 and TK25, and *Saccharopolyspora erythraea*) enabled them to degrade IR [58]. However, inserting *lcp* genes with different plasmids into *Escherichia coli* and *Pseudomonas* bacterial strains was not successful as the *lcp* protein produced was inactive.

Other functional genes present in Actinobacteria, or Gram-positive bacteria might be needed for *lcp* to function, or perhaps the morphological characteristics unique to Actinobacteria are needed to fully express activate *lcp*. Although there are suggestions that the formation of mycelia contributes to the formation of clear zone on NR latex agar plates, we still are yet to understand why Gram-negative rubber degraders also produces clear zone on NR latex agar plates. Some authors speculate that *lcp* can occur in two different conformations, an open and a closed state and adhesively growing bacteria harbors *lcp* in an open conformation, which is accessible to external ligands and substrates without the need for a conformational change [4,49]. This however, contradicts the findings where *Lcp*1VH2 was able to prompt *S. lividans* TK23 (a non-rubber degrading strain) to form clearing zone, while its natural host *G. polyisoprenivorans* VH2 (adhesive rubber degrader) does not produce clearing zone [49]. Similarly, in another study, when native plasmids of *G. westfalica* strain Kb1 containing *lcp* gene were removed, the strain lost the ability to grow on NR as the sole carbon source [59].

*Gordonia polyisoprenivorans* VH2 contains two *lcps*, *lcp*1VH2 is located on the chromosome and *lcp*2VH2 is located on its plasmid p174, and their activity was tested [60]. Deletion of both genes resulted in a non-rubber-degrading phenotype, whereas single-deletion mutants still grew on rubber [60]. Strains that contain more than 1 *lcp* gene in either their chromosome or plasmids are believed to have evolved through their ability to quickly transfer genes (horizontal gene transfer, HGT) and adapt for survivability. The presence of a second *lcp* gene might be needed to improve the organism’s ability to adapt to changing environment by contributing to its ability to utilize different rubber products or increase the rate of degradation.

Several quantitative *lcp* gene expression studies have been carried out using qPCR (real time polymerase chain reaction); *Nocardia* sp. strain NVL3 *lcp* gene showed 1596-fold higher in the presence of IR [61], *Actinoplanes* strain OR16 showed increase of 22.2-fold, 17.1-fold, 335-fold for *lcp*1, *lcp*2 and *lcp*3 gene respectively when cultivated in NR [47], while *Streptomyces* sp. K30 and *G. polyisoprenivorans* VH2 showed expression of *lcp* gene when cultivated with poly-(*cis*-1,4-isoprene) compared to those grown with glucose or sodium acetate [57,62]. *Lcp* was considered a key enzyme found in exclusively in Gram-positive strains, until recently, when the *lcp* gene was discovered in Gram-negative bacteria, *Solimonas fluvinis* [63]. However, the ability of *S*. *fluvinis* to utilize rubber as a nutrient source has not been reported, and further functional studies would be needed to clarify the actual function of *lcp* in Gram-negative bacteria [44].

#### Rubber Degradation by *Lcp* Enzyme

The degradation of rubber by bacterial strains producing *lcp* enzymes results in a variety of degradation products. The range of degradation products obtained can be due to the structural or chemical base components of the rubber material that are degraded, or the presence of *lcp* homologues that differ in their activity. There have also been reports where the combination of *Lcp*K30 and *rox*A or *rox*B has a synergistic effect on the rate of rubber degradation [48]. Therefore, a combination of different *lcp* proteins may also act synergistically and improve the degradation process, this has been shown when a consortium of rubber degrading strains was tested [64]. To have a better understanding of *lcp* functionality, one would need a comprehensive understanding of the rates and mechanisms of *lcp* rubber degradation using excess purified stock of individual enzymes.

### 3.2. RoxA and RoxB

Rubber oxygenase A (*rox*A) and rubber oxygenase B (*rox*B) are key enzymes in NR degradation by Gram-negative bacteria [65]. These groups of bacteria are either less abundant in nature or are infrequently isolated due to the absence of growth factors in the media used for screening purposes [50].

*S. cummioxidans* 35Y (previously known as *Xanthomonas* sp. strain 35Y) was the first Gram-negative rubber degrading bacteria isolated [51,66]. This strain has become one of the best-studied rubber degraders, and its activity causes the formation of oligomers with Mw’s between 10^3^ and 10^4^ Da [51]. *S. cummioxidans* 35Y causes a ~60% weight loss of solid NR pieces within one week of incubation, marking this strain as one of the faster NR-degrading microbes [66]. *Rox*A35Y and *Rox*B35Y (*S. cummioxidans* 35Y) work in a complementary fashion—*Rox*B35Y cleaves polyisoprene to a mixture of C_20_- and higher oligoisoprenoids while *Rox*A35Y cleaves both polyisoprene and *rox*B35Y-derived oligoisoprenoids to the C_15_-oligoisoprenoid, 12-oxo-4,8-dimethyltrideca-4,8-diene-1-al (ODTD) [67]. *Rox*B has only 36% similarity to *rox*A.

Other genera that have been identified to contain *rox*A *or rox*B are *Brevundimonas* sp., *Rhizobacter* sp., *Pseudomonas* sp., *Enterobacter* sp., *Methylobacterium* sp., *Acinetobacter* sp., and *Methylibium* sp. [3,52,68,69,70,71,72,73,74]. Functional *rox*B and *rox*A orthologs, *Lat*A1 and *Lat*A2, respectively, essential for NR degradation by *R. gummiphilus* NS2 has also been reported [67].

*Rox*A-related sequences were also identified from members of the Myxobacteria, namely, *Haliangium ochraceum*, *Corallococcus coralloides*, *Myxococcus fulvus*, and *Chondromyces apiculatus* [74]. However, no rubber degrading activity has been reported. None of the currently genome-sequenced Gram-positive species has a *roxA* or a *roxB* gene [63].

### 3.3. Others: Enzyme Mediator Systems

Although there are only 3 rubber degrading enzymes discovered to date, enzyme mediator systems have been reported to enhance the rate of rubber degradation. In the case of *Microbacteria*, the in vitro oxidative degradation of *trans* and *cis*-polyisoprene based on free radical chain reactions of lipids with different enzymes like manganese peroxidase, laccase, horseradish peroxidase and lipoxigenase/linoleic acid have been reported [75,76]. The results of the enzyme treatments were promising, revealing hole formation in the substrate after just 2–7 days [69]. Fetzner (2002) postulated a reaction mechanism for cofactor-independent dioxygenases in which an amino acid residue (probably histidine) functions as a proton acceptor [77]. A combination of both enzymes and rubber oxygenase may allow a new mechanism that does not require a fatty acid mediator [76].

### 3.4. Unknown: Rubber Degradation in Fungi

Screening of fungi for degrading NR dates back to 1928. However, subsequent reports on rubber degrading fungi were mostly descriptive in nature, only indicating it had the ability to degrade both NR and IR. Rubber degrading fungi genera include *Monascus*, *Aspergillus*, *Penicillium*, *Fusarium*, *Cladosporium*, *Paecilomyces*, *Phoma*, *Calotropis* and *Phlebia* [58]. Endophytic fungi were also found to have rubber degrading properties [78]. Recently, a rubber plant pathogen, *Rigidoporus microsporus* that can grow on latex was sequenced, however, no homologs of bacterial proteins involved in latex degradation were identified in the genome. The analysis of RNA-seq data suggests that *R. microporus* might utilize proteinaceous components of the latex, as higher transcript abundance was found for several genes encoding proteases and transporter proteins [79]. Hence, not all strains that are able to grow in the presence of latex contain rubber degrading genes.

## 4. *Lcp* Gene and Its Pathway

To identify whether the microbial rubber degradation is caused by *lcp* gene, one should amplify the gene and confirm the presence of conserve region. More complete sequencing would reveal *lcp* related genes, such as *oxi*AB genes, TAT secretion motif, and *lcp* regulator, which contribute to the rubber degradation pathway.

### 4.1. Conserved Region

*Lcp* from different species are related in amino acid sequence and share a common domain of unknown function DUF2236 together with other hypothetical proteins with different functional annotations [54,80]. DUF2236 sequences (1280 out of 6000) were analyzed and a 13-residue-long highly conserved region (KTRLVHAAVRHLL) in the primary amino acid sequence between K193 and L205 (in *Lcp*K30) was identified [80].

Histidines are known to associate heme in proteins within this domain. Heme is an essential molecule and plays vital roles in many biological processes. It is evident that the three conserved DUF2236 residues (R164, T168, and H198) were highly conserved in the 495 *lcp* homologues by 98.8%, 99.6%, and 100%, respectively [80].

Three histidines in G. *polyisoprenivorans lcp* 1 (H195, 200 and 228) are highly conserved, and mineralization of rubber showed that histidine at position 195 is essential for strain VH2 to use rubber as sole source of carbon and energy [81]. It was further confirmed that heme-association is dependent on the presence of histidine at position 195 [81]. An analogue for H195 in *Lcp*K30 is located at position 198, exchange of this amino acid (H198 or H195) led to the loss of heme [80].

### 4.2. Lcp Operon & Rubber Degradation Pathway

**Figure 4:**
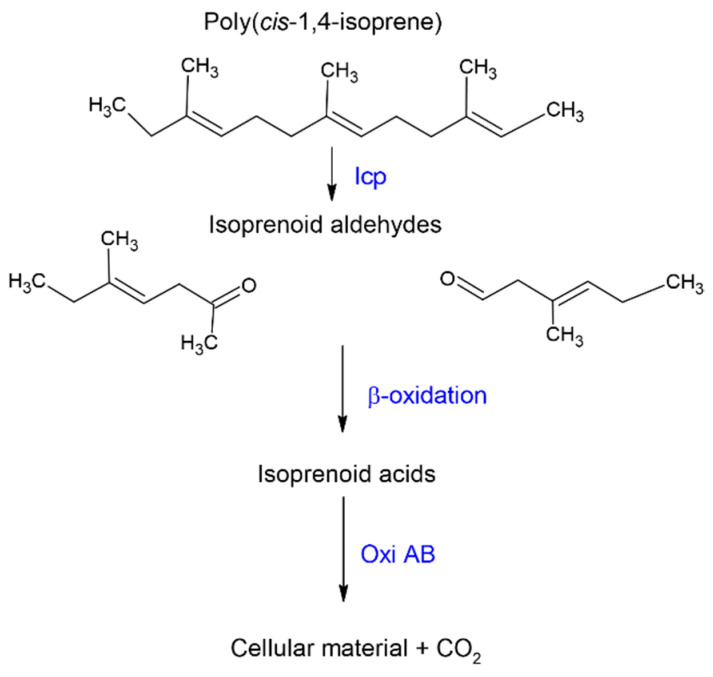
Schematic diagram representing the primary step of *cis*-1,4 isoprene biodegradation.

Although the mechanism of rubber degradation is not fully understood, several authors have postulated a rubber degrading pathway [46,60,82,83]. Here, we highlight the main sections of the putative rubber degradation pathway (Figure 4).

Three genes, namely *lcp*, *oxi*A, and *oxi*B, seem to exist as an operon having a *polyisotronic* mRNA. *Lcp* catalyzes cleavage of the C=C double bond of poly(*cis*-1,4-isoprene) found in NR and IR. In the process, oxygen is added across the double bonds, leading to a mixture of oligonucleotide-isoprenoids with terminal keto and aldehyde groups [1,84]. It is speculated that *lcp* binds and cleaves the polymer while *oxi*A and *oxi*B, located downstream of *lcp*, catabolize the degraded products [69]. The smaller oligomers are subsequently taken up by an Mce-protein (Mammalian cell entry) driven transport mechanism and are converted into the corresponding acids by a putative heterodimeric molybdenum hydroxylase, *oxi*AB, before entering the β-oxidation pathway [7,82]. OxiA is a small subunit critical for the iron-sulfur cluster center, while *oxi*B is a large subunit that possibly acts to bind molybdopterin cytosine dinucleotide cofactor [7]. *Oxi*AB a molybdenum-dependent hydroxylase and is secreted extracellularly. The low molecular weight primary degradation products converted by oxidation to key compounds, including carboxyl groups, can then be taken up and metabolized by the cells via β-oxidation [4,44]. Other rubber degraders, such as *Gordonia* and *Nocardia*, do not possess an *oxi*AB homolog but contain aldehyde dehydrogenase genes (GPOL_c02580, GPOL_c37100) that theoretically perform a similar function [44].

### 4.3. Lcp Secretion Pathway

The *lcp* enzyme is exported by the (twin arginine translocation) Tat secretion pathway-dependent signal motif (RRxxLK) and a cleavage site for the signal sequence (AxA) was found [46]. In contrast to the Secretion (Sec) pathway which transports proteins in an unfolded manner (*rox*A and *rox*B), the Tat pathway serves to actively translocate folded proteins across a lipid membrane bilayer [85]. Folding secreted proteins before translocation would increase their stability and prevent protein aggregation intra- and extra-cellularly [7]. Tat-dependent proteins possess a variety of important functions that have an impact on bacterial cell physiology, such as respiratory energy metabolism, iron acquisition, stress response, and cell division [85].

### 4.4. Lcp Regulator

Two different types of TetR family of regulators (TFRs) were detected upstream of respective *lcp*s for *G. polyisoprenivorans* VH2 (*Lcp*VH2: dimer) and *S. coelicolor Lcp*RBA3(2) (*Lcp*RB: monomer). BLAST analyses revealed sequences coding for homologs of *Lcp*RVH2 among other Actinobacteria, indicating a conserved regulation mechanism of *lcp*s [81], [86]. *Lcp*RBA3(2) belonging, binds the upstream region of *lcp*A3(2) including the palindromic sequence (5′TATGTTAAT-N2-AAAATCACA-3′), to regulate the *lcp*A3(2) transcription, two DNA-binding HTH domains were found in the N-terminal of this protein [44]. Twelve strains that exhibited homolog gene coding for *Lcp*RB and containing *lcp* gene were analyzed. Thereby, fifteen bases exhibited a conservation of at least 90%, including the inverted repeat that was detected in footprinting analyses inside the operator site of *Lcp*RBA3(2) [86]. Notably, the *Lcp*RB sequence was not always located directly next to *lcp*.

Recently, a novel key regulator in poly(*cis*-1,4-isoprene) degradation, cAMP receptor protein (CRPVH2), was detected for *Gordonia polyisoprenivorans* VH2, having 16 bp TGTGAN_6_TCACT motif [87]. CRP is also known as catabolite activator protein, CAP. CRPVH2 and G1xR has 79% sequence identity, both having a common oligomerization state, similar binding motifs and thus most 26 likely similar functions as global regulator [87]. G1xR is a well -studied global regulator of *Corynebacterium glutamicum*.

**Figure 5:**
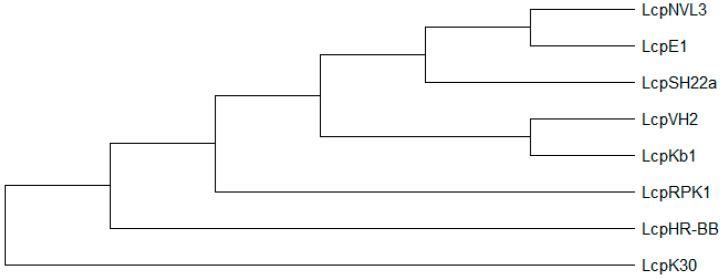
Phylogenetic tree of *lcps* using minimum evolution method was constructed using MEGA X [90]. Enzymes: *Lcp*K30 (AAR25849); *Lcp*1VH2 (ABV68923); *Lcp*Kb1 (ABV68924); *Lcp*SH22a (WP025350295); (AMY60409); *Lcp*NVL3 (API85527); *Lcp*E1 (ABC59140) and *Lcp*HR-BB (WP104231946).

Groups of *Lcp: Lcp* protein characterization has been reported for *G. polyisoprenivorans* VH2, *Lcp*1VH2 [83]; *G. westfalica* Kb1 [62], *R. rhodochrous* RPK1, *Lcp*RPK1 [49]; *Streptomyces* sp. strain K30, *Lcp*K30 [4]; *Nocardia nova* SH22a, *Lcp*SH22a [88]; *Nocardia* sp. strain NVL3, *Lcp*NVL3 [61]; *N. farcinica* E1, *Lcp*E1 [89] and *Solimonas fluminis* HR-BB, *Lcp*HR-B [63]. Phylogenetic analysis of *lcp* proteins showed that *lcp* gene might have evolved separately in each genus (Figure 5) [44]. Hence, the discovery and characterization of novel rubber degrading genera would be interesting and useful to decipher the distribution and characteristics of each *lcp* genes. 

## 5. Latex Clearing Protein: Synthesis

To further understand *lcp* activity and its implications for rubber degradation, we need to express and purify the enzyme. Large scale production of the active enzyme is needed for characterization studies and for industrial application of these enzymes in rubber waste treatment.

The synthesis of active *lcp*1VH2 (*G. polyisoprenivorans* VH2) in *E. coli* was first described by Hiessl et al., (2014). Purification of the protein was performed by immobilized metal affinity chromatography (IMAC) and size exclusion chromatography (SEC) [46]. The poor solubility of *Lcp*1VH2 and the dramatic loss of target protein during the purification process, yielded low amounts of the desired protein. Following this, cultivation in Auto Induction Medium (AIM) and purification using ammonium sulphate precipitation yielded a 30-fold higher concentration of soluble *Lcp*1VH2 (60 mg L^−1^). However, about 90% were inclusion bodies [91]. Using recombinant strains of *E. coli,* high yield production of soluble *Lcp*1VH2 protein (223.5 mg L^−1^) through fed batch fermentations was successfully achieved [56]. Using a defined mineral salt medium [92] for *E. coli* cultivation, the group was able to significantly increase the biomass up to nearly 10-fold and the total *Lcp*1VH2 yield up to 3.7-fold, reducing the costs for the medium by 75%.

R*ox*A was successfully expressed from *S*. *cummioxidans* 35Y with a yield of 15 mg L^−1^ of secreted protein. However, heterologous expression of *rox*A has not been successful to date [93,94].

## 6. Actinobacteria and Their Potential for Biodegrading Rubber

Actinobacteria is the largest group of bacterial rubber degraders identified today. In this section, we discuss the diversity and characteristics of Actinobacteria which make them favorable as rubber degraders. Many reports on the isolation and screening of rubber degrading bacteria have been published. However, with the advancement of genomic sequencing, we compiled a list of rubber degrading Actinobacteria which gives us an insight on the overall distribution of *lcp* genes and isolation source (see Section 6.2).

### 6.1. Diversity

Actinobacteria are one of the largest and most diverse phyla among the bacteria with a well described phylogeny. According to the latest classification, phylum Actinobacteria is divided into 6 classes, namely: Actinobacteria, Acidimicrobiia, Coriobacteriia, Nitriliruptoria, Rubrobacteria, and Thermoleophilia [95]. The class Actinobacteria is further divided into 16 orders that are Actinomycetales, Actinopolysporales, Bifidobacteriales, Catenulisporales, Corynebacteriales, Frankiales, Glycomycetales, Jiangellales, Kineosporiales, Micrococcales, Micromonosporales, Propionibacteriales, Pseudonocardiales, Streptomycetales, Streptosporangiales, and *Incertae sedis* [95]. The genera of this class exhibit enormous diversity in terms of their morphology and physiology capabilities. Morphologies range from coccoid (e.g., *Micrococcus*) or rod-coccoid (e.g., *Arthrobacter*) to fragmenting hyphal forms (e.g., *Nocardia* sp.) or permanent and highly differentiated branched mycelium (e.g., *Streptomyces* sp.) [96]. Diverse metabolic properties, such as the production of extracellular enzymes and the formation of a wide variety of secondary metabolites, are also exhibited in this group [97,98].

### 6.2. Distribution

Actinobacteria members have adopted diverse lifestyles; they may be inhabitants of soil or aquatic environments (e.g., *Streptomyces*, *Micromonospora*, *Rhodococcus*, and *Salinispora* species), pathogens (e.g., *Corynebacterium*, *Mycobacterium*, *Nocardia*, *Tropheryma*, and *Propionibacterium*), solely soil inhabitants (*Streptomyces*), plant commensals (*Leifsonia*), plant symbionts (e.g., *Frankia* spp.), or gastrointestinal commensals (*Bifidobacterium*) [99]. The main microbial population is found in the surface layer of soil (10^6^–10^9^ cells gram^−1^), where they play a crucial role in recycling refractory biomaterials by decomposition and humus formation [100,101]. Common genera in soil samples are *Streptomyces* (nearly 70%), *Nocardia*, and *Micromonospora*, although *Actinoplanes* and *Streptosporangium* are also generally encountered [102]. They live under the most diverse conditions, aerobic and anaerobic, at temperatures of 5–7 °C and 45–70 °C [103]. Thermophilic strains (e.g., *Thermoactinomycetes*, *Streptomyces*) are important for biotechnological investigations and the ability to grow at high temperatures makes them suitable candidates for rubber biodegradation. Thermostable enzymes are more resistant to industrial processes with high solvent or high salt concentrations and offer potentially faster reaction kinetics, and importantly, high temperatures in a fermentative process to prevent costly microbial contamination [104].

### 6.3. Genomic Characteristics of Actinobacteria

Actinobacteria have high G+C base content, ranging from 51% in some *Corynebacteria* to more than 70% in *Streptomyces* and *Frankia* [99]. However, the G+C content is less than 50% in *Gardnerella vaginalis* and *Tropheryma whipplei*. Of those analyzed, all Actinobacteria have a single chromosome, but the presence of large plasmids is not uncommon. Base composition is a fundamental property of genomes having a strong influence on gene function and regulation [105]. Bohlin et al. (2017) found that the coding regions in core genomes are significantly more GC-rich [106].

The genome content in bacteria is shaped by the interplay between vertical inheritance (clonal reproduction) with gene loss and acquisition, the latter either involving the genesis of new genes within a lineage or horizontal gene transfer (HGT) from other lineages [96]. Microbial genomes evolve dynamically by both losing and gaining genes, to create new species. HGT is held responsible for enhancing the competitiveness of bacteria in their natural environments [98]. Some Actinobacteria strains have a linear chromosome, whereas most of the genera harbor a circular chromosome, or undergo chromosomal circularization [107,108,109]. Actinobacteria with a more complex life cycle and structure, such as *Nocardia*, *Actinoplanes*, *Micromonospora*, *Streptovericillium*, *Streptomyces*, and *Saccharopolyspora*, generally have a linear chromosome [110,111,112].

In general, the chromosomes found in Actinobacteria are large, relative to other bacteria, with an average size of over 5 megabases (Mb) [98]. However, genome sizes vary widely: from less than 1 Mb in some *Tropherma* to over 12 Mb in some *Streptomyces*. The microbial genome or pan genome consists of a “core” section, representing about half the genome, and “accessory” elements including the ends of the linear structure [105,113]. Essential genes lie within the core while genes whose products would be expected to be adaptive tend to be present in the arms [110,114]. Gene inactivation and loss are common in groups with a host-associated lifestyle; the host supplies many of the metabolic intermediates, thereby eliminating their need to maintain many biosynthetic genes [98]. Gross chromosomal rearrangements are ubiquitous among *Streptomyces* and affects nearly all life functions, e.g., differentiation, secondary metabolism, and response to environmental changes [115].

Plasmids (extrachromosomal DNA) can be divided into five main types: fertility F-plasmids, resistance plasmids, virulence plasmids, degradative plasmids, and finally Col plasmids [116]. They carry genes that usually confer beneficial properties and are required for the survival of the host. One of the unique features of Actinobacteria, especially the genus *Streptomyces*, is the presence of linear plasmids, sized from 12 to 600 kb, often termed mega-plasmids [117].

### 6.4. Latex Clearing Protein in the GenBank: Actinobacteria

The GenBank protein database recorded 248 Actinobacteria with a DUF2236 domain containing latex clearing protein (accessed April, 2020). These strains consist of 16 families, 29 genera and 134 species (Table 2). Most of the genes were from *Streptomyces* (20.6%), followed by *Mycobacteriodes* (17.4%), and *Nocardia* (11.7%). When we profile the 248 *lcp* genes using a phylogenetic approach, 12 distinct clusters were observed, suggesting that there may be 12 different groups of *lcp* genes (Supplementary Figure S1). By identifying the *lcp* groups, we could screen for *lcp* genes instead of using living cultures which are limited by screening method used. This is made easier to screen as most culture collection centres have started making DNA extract available upon request.

Within the groups, *lcp* genes seem to be closely related to the strain taxonomy, however, the genera can also be distributed among other clusters. *Lcp* of the same genera within the clusters could be due to gene duplication, or *lcp* genes from different genera within the same cluster could be contributed to gene transfer. 

Jendrossek et al. (1997) screened 1220 Actinobacteria strains, where the majority of the rubber degrading strains (50 strains in total) belonged to *Streptomyces* group (33 strains, 66%) [50]. *Streptomyces* is a genus well known for its ability to secrete enzymes, which can metabolize or cleave biopolymers [8]. Interestingly, strains containing the second and third most *lcp* genes belonged to the mycolic acid producing genera (*Mycobacteriodes* and *Nocardia*).

The number of published family for Actinobacteria is 45 and 306 genera, leaving many more potential rubber degrading genera yet to be discovered [118,119]. Among genera that could be of interest are thermophilic and mycolic acid strains such as *Rhodococcus*. In Table 2, 15 *lcp* genes from nine species were identified from *Rhodococcus* genus. To date, only two *Rhodococcus* strains able to degrade rubber have been isolated; *R. rhodochrous* strain RPK1 from a waste pond at a rubber processing factory in Thailand [50] and *R.* *pyridinivorans* strain F5 from samples collected from various contaminated rubber sites in Songkhla, Thailand [65]. *Rhodococcus* is a promising genus for rubber degradation as they are fast growers and easily cultivated. They have remarkably catabolic versatility, being able degrade an impressive array of xenobiotic and organic compounds. Presently, there are over 1800 and over 3200 patents retrieved for strains of this genus (Google patent search) respectively, as keywords [120].

Thermotolerant Actinobacteria such as strains of *Streptomyces albus* and *Streptomyces griseus*, and thermophilic Actinobacteria such as *Streptomyces*, *Amycolatopsis*, *Microbispora*, *Cellulosimicrobium*, *Micrococcus*, *Saccharopolyspora*, *Micromonospora*, *Thermobispora*, *Thermomonospora*, *Thermobifida*, and *Planomonospora* were reported to be involved in the composting process [121]. A thermotolerant oil degrading Actinobacteria from the genera *Rhodococcus* and *Gordonia* showed ability to grow at up to 10% NaCl and utilize crude oil and individual hydrocarbons at higher (up to 50 °C) temperatures [122]. However, all rubber-degrading species described so far are mesophilic, with only one exception, identified as a *Streptomyces* sp. strain La 7, able to grow at 55 ˚C [123]. Eight moderate thermophilic strains (50 °C) were isolated and tentatively identified as *Actinomadura* sp.*, *Nocardia farcinica*, and *Thermomonospora curvata* (blast homology 96.5*–99.9%) [89].

Among the deposited Actinobacteria strains containing *lcp* genes, 86 of them were isolated from various sources across the world, mainly from soil samples (57%), plant samples (20%), clinical samples (14%), water samples (7%), and animal/ insect samples (2%). Clinical samples mainly consist of *Nocardia*, *Mycobacterium*, and *Mycobacteroides* strains (Supplementary Table S1). *Lcp* containing strains were also isolated from plants that do not produce latex, such as wheat plants and oak trees.

The conserved region for the strains isolated from various sources showed high similarity to the region reported by Rother et al. (2016) (KTRLVHAAVRHLL) (see Section 4.1) except for a sample from animal origin (RVRLIHGLVRKHV) [80]. The strain *Mycolicibacterium malmesburyense* (CRL78180) was isolated from cattle *(Bos Taurus*) in South Africa. When comparing the 248 sequences from these samples, there are other highly conserved regions to be further examined (Supplementary Figure S2). Further investigation of these conserved regions would likely identify novel functions attributed to these strains.

## 7. Rubber Biodegradation

Researchers have been studying microbial rubber degradation since the early 1900s. In this section, we discuss the main methods used to conduct and evaluate rubber degradation under laboratory conditions and possible ways to apply rubber biodegradation in our current regime.

### 7.1. In Vivo Rubber Degradation Using Microbial Culture

In the laboratory, rubber degradation studies are carried out by incubating the microorganism with rubber materials (0.5–0.6% *w*/*v*) supplemented with mineral salts medium (MSM) on a shaker for 6–12 weeks at 30 °C [68,124]. Examination of the size, final mass, and morphology of the rubber materials after incubation indicates the relative activity of the microorganism in question. Alternatively, the bacteria are inoculated on NR latex (concentrated and purified) overlay agar plates, consisting of a bottom agar layer of MSM and incubated for 3–7 days at 30 °C. Their ability to form clear zones on the NR latex layer are then evaluated.

Table 3 shows the rate of rubber degradation for some strains. Among the parameters recorded were duration, percent weight loss, material tested and pre-treatment before introducing the bacteria. Most of the studies were carried out at 150 rpm shaking. Separate observations showed the unfavorable effects of agitation on the rate of rubber degradation. For example, the formation of biofilms could be limited due to constant agitation. *Nocardia* sp. 835A-Rc, a mutant strain, uniformly attacked tyre particles when stirred at 0–40 rpm. However, higher stirring rates led to some clumps of microbial colonies on the rubber surface and separate deep semi-spherical cavities [125]. Using *G. polyisoprenivorans* VH2 culture, Berekaa and his colleagues (2000) tested six weeks of shaking, followed by six weeks of stationary incubation, which led to the complete disintegration of the rubber material [124].

Pre-treatment of samples to remove impurities, composites materials and antimicrobial substances improved colonization efficiency and degradation rates [124]. The impurities removed by pre-treatment can otherwise leach into the surrounding media during long cultivation periods and may be unfavorable to microbial growth. Constant replacement of MSM media also enhanced the rates rubber disintegration which then led to complete visual loss of the rubber material at week 10 [124].

The addition of a starch component to the incubation media also improved degradation. The starch is metabolized first and acts as a growth substrate for rubber degrading bacteria, to increase initial bacterial populations rapidly, and subsequently the rate of NR degradation [127,128,129,130,131].

Actinobacteria has also showed their ability to degrade IR breaking cross-link Sulfur-Sulfur or Carbon-Sulfur bonds with the main chain remaining intact. *Streptomyces* sp. was able to devulcanize IR after 4 weeks incubation, having 18.13% of carbon loss and 52.90 reduction in cross linking degrees [132]. *Streptomyces coelicolor* 1A was able to degrade IR (Aldrich, M_w_ 800,000), having 9% of IR detected with M_w_ less than 10,000 after six weeks [68]. *Gordonia amicalisa* was able to reduce vulcanized IR and vulcanized styrene butadiene rubber crosslink densities by 13.7% and 22.1%, respectively, vulcanized styrene butadiene rubber sulfur content on its surface was decreased by 22.9%, S-S bonds were broken, producing S=O bonds [133]. *Amycolaptopsis sulphurea* DSMZ 46092, *Gordonia* DSMZ 44215 and *Gordonia* DSMZ 44369 showed 20.90%, 18.64%, and 17.52% respectively showed decrease in sulfur after 4 weeks of incubation [134]. In patent US 7737191B2, mycolic acid containing Actinobacteria strains of *Corynebacterium*, *Rhodococcus*, *Nocardia*, *Gordonia*, *Tsukamurella*, *Dietzia*, and *Mycobacterium*, and mainly *Gordonia desulfuricans* strain SG213E were used for rubber treatment [135]. 

These findings show the potential of Actinobacteria to degrade both NR and IR in treatment of rubber wastes.

#### 7.1.1. Measurements for Rubber Degradation

The efficiency of bacteria in degrading rubber is evaluated based on the organism’s effect on several stages of biodegradation: biodeterioration, biofragmentation, bioassimilation and mineralization. Scanning electron microscopy (SEM) is mainly used to observe the degree of deterioration that the rubber material undergoes following exposure to the organism. In the main, this is a qualitative measure that looks for changes in the surface of the rubber.

In biofragmentation, the original polymer is fragmented resulting in fewer *cis*-1,4 double bonds, and the formation of new carbonyl groups. These chemical changes are usually detected using attenuated Total Reflection-Fourier transform infrared (ATR-FTIR) spectra. Based on the ATR-FTIR profile for NR, changes in ~873 cm^−1^ indicate change of C=H bond, double peak at 1250–1500 cm^−1^ refers to the presence of amide I and II, while at ~1715 cm^−1^ peak refer to C=O bond, and ~3400 cm^−1^ peak refer to O=H bond [136]. Presence of aldehydes containing oligomers can also be tested by staining the rubber material using Schiff’s reagent, resulting in purple coloration.

Differences in rubber molecular weight before and after bacterial inoculation, can be measured using Gel Permeation Chromatography (GPC). Weight loss measurements indicate that the bacteria are able to cleave the carbon backbone of NR or IR and utilize the low molecular-weight degradation products for growth. Polymer without microbial inoculation (control) shows Gauss-like distribution of molecular weights between 10^4^ and 10^7^ (Mw, 8 × 10^5^) [68]. 

The rate of rubber mineralization under aerobic condition can be calculated by measuring the amount of CO_2_ released during the cultivation of cells with rubber materials in tightly closed Erlenmeyer flasks over time. The released CO_2_ is trapped as BaCO_3_, from reaction with Ba(OH)_2_, which is subsequently processed to determine the CO_2_ content [127].

For IR, additional measurements can be carried out. Scanning Electron Microscopy (SEM) coupled to Energy Dispersive Spectroscopy (EDS) will enable comparison of EDS maps for carbon (C), oxygen (O) and sulfur (S) with control samples [132]. Horikx analysis determines the breakdown in a vulcanized rubber network by the rate of increase of the soluble fraction of the rubber as a function of the measured cross-link density of the remaining total insoluble fraction is different for cleavage of S-S and C-S bonds [137]. Crosslinking degree measurements can be determined by boiling 20 mg of vulcanized rubber in a tightly closed filter paper at 110.6 °C to a leaching system for 48 h, the crosslinking degree, related to the insoluble residue, was calculated as the ratio between the weights of the sample after and before being soaked in toluene during the leaching process [132].

#### 7.1.2. Drawbacks

The quantitative measurement of in vivo rubber degradation using culture is limited and the results from studies on different strains are often difficult to compare. The presence of clearing zones, rates of growth, and zone sizes on NR latex overlay agar plates are affected by various parameters such as strain type, growth rate, strain age, amount of strain inoculated (a drop, a colony etc.). Several of the most potent rubber degraders (*G. polyisoprenivorans* and *G. westfalica*) do not produce clearing zones and requires close contact with the rubber material they degrade. Methods employed for rubber degradation studies are also inconsistent between different research groups (type, size and thickness of rubber material, rubber material pre-treatment, number of days, amount of inoculum, agitation rate etc.). The surface characteristics of the tested material are crucial as high surface ratio (smaller particles) and the presence of rough rubber surfaces and cracks are favourable for microorganism growth and attachment [138]. SEM observation showed that *Paecilomyces variotii* strain SFA-25 hyphal network and rubber surface erosion was more intense on rough and cracked surfaces compared to smooth surfaces.

Rubber degrading strains may contain more than 1 *lcp* gene, combination of the gene products may have synergistic activity; alternatively, the products may be dysfunctional. Further investigations into the functions of additional genes, especially in the chromosome, are needed to understand how these *lcp* genes may or may not interact to degrade rubber. Three types of rubber oxygenases for the enzymatic cleavage of rubber has been tested using RoxAXsp, RoxBXsp, *Lcp*K30 or with combinations of the three proteins resulting in differed in the number of intact isoprene units, having the same general structure with terminal functions (CHO-CH_2_- and -CH_2_-COCH_3_) [48].

### 7.2. In Situ Rubber Degradation

Several possible strategies for using Actinobacteria for in situ rubber degradation are discussed here: bioaugmentation, biotransformation, bioreactors, and synthetic biology. There is a strong possibility that combining these strategies with the appropriate strain could be applied for use at larger scale to help reduce rubber pollution in the environment.

#### 7.2.1. Bioaugmentation and Consortia

Biomimicry can improve the degradation process through introduction of efficient microbial strains (bio-augmentation) or by addition of nutrients to the soil (bio-stimulation) [139]. Bioaugmentation is a technique for improving the degradative capacity of contaminated areas through introduction of specific competent strains or consortia of microorganisms (autochthonous or allochthonous wild type or genetically modified microorganisms, GEMs) [140].

The main limitation of using bioaugmentation for rubber wastewater treatment is the microbial survival rate. Several potential solutions can be explored such as increasing the amount of inoculant or incorporation of inoculant by time-based, addition of nanomaterials to support the organism, and the of GEMs to improve survival [141].

Bio-stimulation refers to the addition of growth-rate limiting nutrients like phosphorus, nitrogen, oxygen, electron donors to severely polluted sites for the enhancing the indigenous biomass activity to degrade the hazardous and toxic contaminants [139]. The primary advantage of bio-stimulation is that bioremediation will be undertaken by already present native microorganisms that are well-suited to the subsurface environment and are well distributed spatially within the subsurface [142].

Recently, the use of monoculture, co-culture and consortium in rubber degradation studies, mimicking the natural environmental condition, has been compared [55,64,72,73,142,143]. The degradation of complex molecules is achieved through co-operative degradation where one organism transforms the original material into products that can be utilized by the other organisms [55,144]. The synergetic interaction of several strains of microorganisms in rubber degradation activity was better when compared to individual strains. This strategy where bacterial strains are combined to enhance the overall degradation of rubber is worth further investigation to understand what combinations work best.

#### 7.2.2. Biotransformation

Microbial fermentation has the potential for making three renewable rubber intermediates: isoprene, isobutene, and butadiene [20]. However, these processes must be cost competitive with the petrochemical pathways. The enzyme isoprene synthase has been identified in plants and through synthetic biology its expression has been optimized in several microorganisms. Andler et al. (2019) proposed a recycling method for rubber waste through biotransformation [145]. Carbon sources obtained from the biodegradation of rubber were used to produce Polyhydroxyalkanoates (PHAs) through recombinant strains of *G. polyisoprenivorans* VH2 harboring plasmid pAK68 (*phaCAB* from *Ralstonia eutropha*) and pAK71 (*phaC1* from *Pseudomonas aeruginosa*). PHAs are environmentally friendly, biodegradable alternatives to petroleum-based plastics made by a group of bacterial polyesters.

There has been success in improving rubber degradation by using a mutant strain *Nocardia* 835A strain Rc, which caused 81% weight loss to tyre thread after eight weeks and its activity was influenced by the NR content [69].

#### 7.2.3. Bioreactors

The use of bacterial strains in rubber wastewater treatment is considered an ecologically and environmentally favorable technology, it provides many potential advantages, such as low energy consumption, ease of the process, low equipment requirements, and no pollution [146]. Stevenson et al. (2008) and other scientists recommend a multistage process utilizing several different microbes and biochemical pathways [147]. Here, the detoxification of scrap tyre rubber (e.g., fungus *Recinicium bicolour*) is followed by sulfur-oxidization (e.g., bacteria *Thiobacillus ferroxidans* or sulfur-reducing archaeon (*Pyrococcus furiosus*). They propose the usage of Actinobacteria for rubber mineralisation and catabolism based on the production of *lcp* and *rox* enzymes after the waste materials have been detoxified and devulcanized. Actinobacteria strains such as *Rhodococcus rhodochrous*, *Arthrobacter sulfureus*, *Gordonia rubropertincta*, and *Rhodococcus erythropolis* can also play a role in desulfurizing [148]. The most common biodesulfurization pathway reported to date is the 4S pathway discovered in *Rhodococcus erythropolis* IGTS8 [149].

#### 7.2.4. Synthetic Biology

Actinobacteria demonstrate many advantages as a rubber degrader, however they can be further improved for application purposes, whether in the industrial process or the environment through synthetic biology. Synthetic biology uses a pared-down life form to serve as a chassis on which to build something with a useful application to humankind such as biodegradation, recycling, waste clean-up, etc. [150]. Only ten microbes have been “domesticated” for industrial use [151]. Synthetic biologists develop their projects through standard engineering cycles of ‘design, build, test’ [150]. The design phase involves computer modelling of the components’ behavior. The build stage involves the genetic engineering. The test step assesses whether the prospective approach works.

Cell-free protein synthesis is another powerful flexible bottom-up approach utilizing a minimum of cellular elements that allows for labor- and time-efficient protein expression in a test tube without multistep complex maintenance of a living culture. The move to cell-free synthesis can increase reaction rates 100-fold [152]. Karzbrun et al. (2014) assembled two-dimensional DNA compartments fabricated in silicon capable of metabolism, programmable protein synthesis, and communication as a powerful route for flexible and controllable production systems [153]. This approach can be used to access non-natural materials and circumvents the potential issue of substrate toxicity to cells (as well as the regulatory issues of genetically modified cells [151].

Building blocks for IR (such as isoprene) are currently sourced from petroleum. Synthetic biology would reduce the cost, carbon footprint of tyres, and most importantly, addresses the issues of microplastics pollution in the environment. Scientists in China have altered a marine bacterium *Synechococcus elongatus* photosynthesis pathway to produce high quantities of isoprene in the laboratory, while researchers in the US are using yeast to convert plant carbohydrates into low-cost alcohols and later converted into isoprene [154,155].

#### 7.2.5. Drawbacks

Rubber biodegradation has yet to be applied outside laboratory testing or at a large scale. Small scale studies involving soil, surface water and groundwater, showed that introduced microorganisms are unable to survive in rubber liquid wastes. There may be other unknown limitations in biodegradation using living cultures or enzymes that we have yet to discover.

There is, however, one successful full scale bioaugmentation story that has been reported is the in *in-situ* removal of chlorinated solvents in ground water, with the use of anaerobic bacteria of *Dehalococcoides* group [156].

In rubber biodegradation, Actinobacteria has many of advantages however one must be aware that there are strains which pathogenic towards animals (*R. equi*), plants (*R. fascians*), and humans (*N. farcinia*, *R. equi*, *R. rhodochrous*, and *R. erythropolis*). In situ application of living strains and GEMs would require environmental impact assessment and further evaluation.

### 7.3. Rubber Reclamation of Tyres by Microbes

Currently, there are vast tyre landfill areas to be addressed, and we can start improving the rate of tyre degradation by introducing beneficial microorganisms including rubber degrading strains to these areas. One must start by enriching and acclimatizing the culture to the landfill or providing suitable starter material, like techniques used in composting. The existing tyres must be reduced in size and pretreated in order to facilitate microbial degradation.

In addressing rubber waste problems, we must also find ways to make rubber products such as tyres using materials that are easier to degrade. Researchers found that using ruthenium to generate cyclopentene as a synthetic rubber material are completely degradable at lower temperature (40–50 °C) [157]. Michelin has proposed the usage of 3-D printing using biodegradable materials for their tyre production [158]. Goodyear recently unveiled a tyre concept ‘reCharge’ using dandelion rubber and fibers that can generate tyre thread as needed [159]. Biodegradation of these materials can then be more feasible in an industrial context (recycling facility, bioreactor) or at landfills.

## 8. Concluding Remarks

In this present review, we have highlighted the incredible utility of rubber in the production of a diverse range of commercial products. Actinobacteria is a group of microorganisms that have the potential to be developed as part of an industrial solution for the biodegradation of NR and IR. Actinobacteria are present in a diverse range of environments, indicating their ability to adapt to local surroundings and thus a survival advantage in withstanding industrial conditions. Of more importance, the strains of Actinobacteria that express dioxygenases which are implicated in the degradation of rubber. Although the enzyme activities were discovered decades ago, it is only with the convergence of genomic screening with functional tests that the distribution and evolutionary patterns of the genes responsible for the enzymes are being elucidated. Molecular techniques are now being used to confirm the mechanisms of action behind rubber degradation by these organisms. Our present understanding of how the dioxygenases act on rubber substrates, and what bacterial, molecular, or environmental factors best support their activity is limited, and it will be crucial to continue screening for novel strains or gene products in tandem with functional assays to fully understand the mechanistic steps behind rubber degradation by the Actinobacteria, and the range of activity they are capable of.

Identifying the critical steps and environmental or molecular factors in the degradation of rubber by Actinobacteria will allow the rational design of bio-processes that can be used in industrial settings. Synthetic biology will be important for producing enzyme stocks, identifying enzyme combinations that degrade rubber synergistically, or integrating active components onto carriers that are designed to protect the active components from harsh industrial conditions. In conclusion, identifying strains of Actinobacteria that degrade rubber is the first step in developing processes for bio-degrading rubber tyres. The second step is to understand the steps and components that play pivotal roles in the process, and to use this knowledge to design bio-systems that can be scaled up and used under industrial conditions.

## Figures and Tables

**Table 1:** Summary of rubber oxygenase’s main characteristics [54,55,56].

	*Lcp*	*Rox*B	*Rox*A
First Identified From	*Streptomyces* sp. strain K30	*Steroidobacter cummioxidans* 35Y (*Xanthomonas* sp. 35Y)	
Accession no.	AY387589	KY498024	KC980911
Metabolite Products	Tetra C_20_, oligoisoprene/ aldehyde and ketone terminal		ODTDs
Bacteria	Mostly Gram-positive	Gram-negative only	
Gene Length (bp)	~1224–1227	~2037–2046	
Size (kDa)	43	73–74	
Pathway	TAT	SEC	
Co-factor	b-type heme	c-type heme	
Mechanism to Cleavage Isoprene	Endo-type	Endo and Exo-type	
Major Metal Atoms in Protein Molecule	1 Fe	2 Fe	
Oxidation State of Iron	Fe^3+^	Fe^3+^----O_2_	

**Table 2:** Diversity of Actinobacteria with DUF2236 domain containing *lcp* genes in GenBank database.

Family	Genus	Genus	Species	*Lcp* Genes	Species Diversity
Actinosynnemataceae	1	*Actinosynnema*	2	3	2
Corynebacterineae	1	*Williamsia*	3	6	2
Frankiaceae	1	*Frankia*	3	7	2
Gordoniaceae	1	*Gordonia*	12	23	2
Microbacteriaceae	1	*Microbacterium*	3	3	1
Micrococcaceae	1	*Psychromicrobium*	1	1	1
Micromonosporaceae	2	*Actinoplanes*	2	4	2
		*Micromonospora*	4	7	2
Mycobacteriaceae	3	*Mycobacterium*	3	6	2
		*Mycobacteroides*	5	43	9
		*Mycolicibacterium*	5	5	1
Nocardiaceae	2	*Nocardia*	12	29	2
		*Rhodococcus*	9	15	2
Nocardioidaceae	2	*Mumia*	1	3	3
		*Nocardioides*	4	4	1
Nocardiopsaceae	2	*Streptomonospora*	1	2	2
		*Nocardiopsis*	1	1	1
Pseudonocardiaceae	7	*Actinoalloteichus*	1	1	1
		*Amycolatopsis*	11	16	1
		*Kibdelosporangium*	1	2	2
		*Kutzneria*	1	1	1
		*Prauserella*	1	2	2
		*Pseudonocardia*	1	1	1
		*Saccharomonospora*	1	1	1
Streptomycetaceae	1	*Streptomyces*	39	51	1
Streptosporangiaceae	2	*Nonomuraea*	1	1	1
		*Streptosporangium*	1	1	1
Thermomonosporaceae	1	*Actinomadura*	2	2	1
Tsukamurellaceae	1	*Tsukamurella*	3	7	2
TOTAL	29		134	248	

Note: Species diversity = number of number of genes divided by number of species.

**Table 3:** Compilation of in vivo culture testing on NR degrading.

Culture	Duration (Weeks)	Weight Loss (%)	Material	Pre-Treatment	Reference
*Nocardia* sp. strain 835A	8	100	NR	–	[45]
		90	Latex glove	Acetone & CHCl_3_	
*S. cummioxidans* 35Y	1	60	NR	NS	[51]
*Amycolatopsis* S1A	6	11	Rubber coated slides (absence of organic nutrients)	Soxhlet purification	[126]
*Amycolatopsis* S1D		12			
*Nocardia* S3F		13			
*Streptomyces* S1G		44			
*Streptomyces* S3D		21			
*Streptomyces* S4C		26			
*Streptomyces* S4D		43			
*Streptomyces* S4E		37			
*Streptomyces* S4F		43			
*Streptomyces* S4G		38			
*S.* sp. strain LA7	8.6	80	Emulgated latex	Dialysed latex	[123]
	3.3	60			
	10	29.4	Latex glove	Acetone & CHCl_3_	
	10	31.3	Latex glove + Triton X (0.1%, *w*/*w*)	Acetone & CHCl_3_	
*S. coelicolor* CH13	6	14	Latex glove	–	[127]
		92	Latex glove + starch (65%)	NS	
*Nocardia* sp. strain 385A	10	5.5	Fresh latex	NS	[128]
*Bacillus cereus*	2	3.5	Tyre	NS	[129]
		7.8	NR	Acetone	
		12.3	Latex film of MR + metroxylan sago pith waste		
*R. pyridinivorans* strain F5	4	9.36	Latex glove	–	[64]

NS: not stated, –: none

## Supplementary Materials

The following are available online at https://www.mdpi.com/article/10.3390/polym13121989/s1, Figure S1: Phylogenetic clusters (12) of 248 *lcp* genes obtained from GenBank, Figure S2: Conserved regions of 248 *lcp* amino acid sequences from GenBank. Sequences were viewed using Jalview, dark purple shades indicate highest percentage identity among sequences, Table S1: Details of Isolation Source for Actinobacteria containing *Lcp* Gene in GenBank.
