# Erratum: Basik et al. Microbial Degradation of Rubber: Actinobacteria. *Polymers* 2021, *13*, 1989

**DOI:** 10.3390/polym13162700

**Published:** 2021-08-13

**Authors:** Ann Anni Basik, Jean-Jacques Sanglier, Chia Tiong Yeo, Kumar Sudesh

**Affiliations:** 1 Ecobiomaterial Research Laboratory, School of Biological Sciences, Universiti Sains Malaysia, Gelugor 11800, Penang, Malaysia; annbasik@gmail.com; 2 Sarawak Biodiversity Centre, Km. 20 Jalan Borneo Heights, Semengoh, Kuching 93250, Sarawak, Malaysia; jjsanglier.esperanza@gmail.com (J.-J.S.); cyeo@sbc.my (C.T.Y.)

The authors wish to make the following changes to the published paper [1] as listed below.

In the original manuscript, Figure 4:

1. Chemical structures for poly(*cis*-1,4-isoprene) were wrongly labelled and have been corrected as shown below.
2. ß-oxidation should be replaced by oxiAB.
3. oxiAB should be replaced by ß-oxidation.

**Figure 4:**
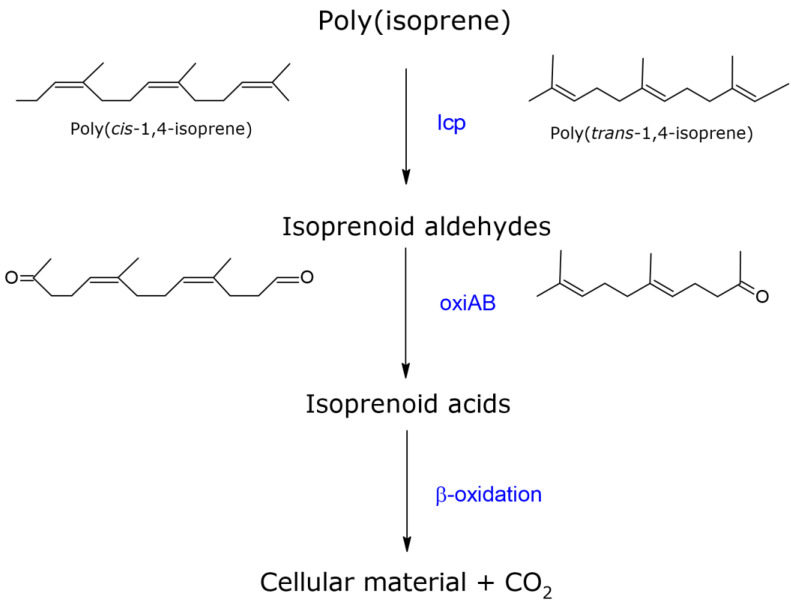
Schematic diagram representing the primary steps of poly(isoprene) biodegradation, followed by oxidization for aldehydes to the corresponding acids, which can be further metabolized via ß-oxidation. Abbreviations: lcp, latex clearing protein; oxiAB, isoquinoline 1-oxidoreductase subunit alpha and beta.
