# Supplementary material for: Microbial Degradation of Rubber: Actinobacteria
Source: Polymers (Basel). 2021 Jun 17;13(12):1989. doi: 10.3390/polym13121989 (PMC8235351; doi:10.3390/polym13121989)
Supplement: Supplementary file 1 [file polymers-13-01989-s001.zip › Supplementary Figure 1.pdf]

Supplementary Figure 1. Phylogenetic clusters (12) of 248 *lcp* genes obtained from GenBank

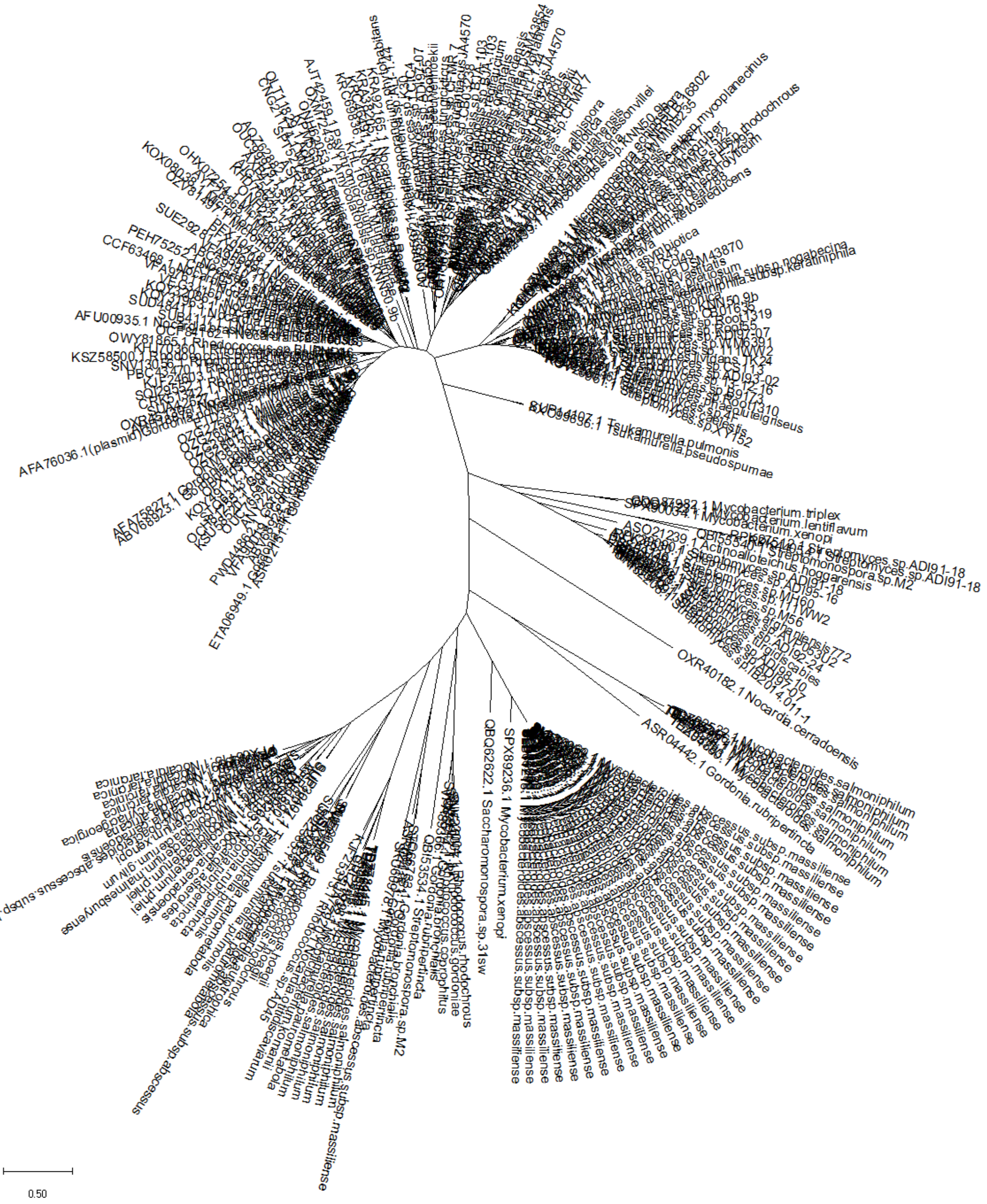

Evolutionary analysis by Maximum Likelihood method

The evolutionary history was inferred by using the Maximum Likelihood method drawn to scale, with branch lengths measured in the number of substitutions per site. This analysis involved 248 amino acid sequences. There was a total of 494 positions in the final dataset. Evolutionary analyses were conducted in MEGA X.
