# Supplementary material for: Microbial Degradation of Rubber: Actinobacteria
Source: Polymers (Basel). 2021 Jun 17;13(12):1989. doi: 10.3390/polym13121989 (PMC8235351; doi:10.3390/polym13121989)

Supplementary Figure 2. Conserved regions of 248 *lcp* amino acid sequences from GenBank. Sequences are viewed using Jalview, dark purple shades indicate highest percentage identity among sequences.

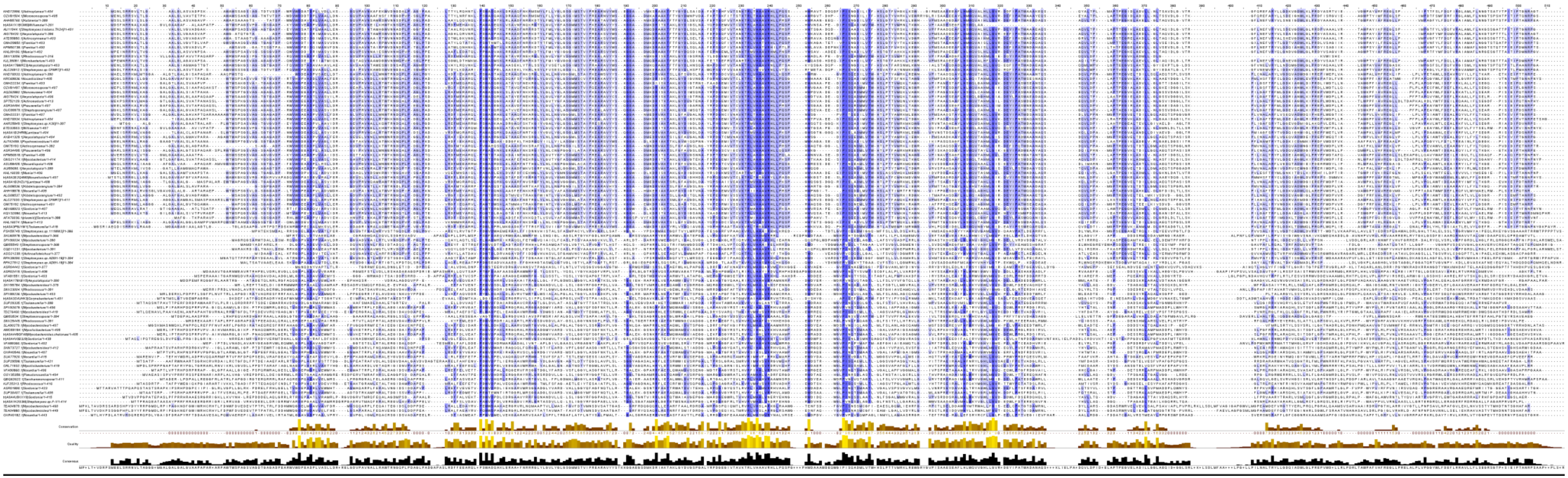

Supplement: Supplementary file 1 [file Supplementary Figure 2.pdf › Supplementary Figure 2.pdf]
