# Supplementary material for: Microbial Degradation of Rubber: Actinobacteria
Source: Polymers (Basel). 2021 Jun 17;13(12):1989. doi: 10.3390/polym13121989 (PMC8235351; doi:10.3390/polym13121989)
Supplement: Supplementary file 1 [file polymers-13-01989-s001.zip › Supplementary Table 1 new.pdf]

Supplementary Table 1. Details of Isolation Source for Actinobacteria containing *lcp* gene in GenBank

GenBank Accession Number and strain	Genus	Country	Isolation Source	Source Group
CRL78180 | Mycolicibacterium malmesburyense	*Mycolicibacterium*	South Africa	Bos taurus	animal
ONI52208 | Streptomyces sp. IB2014 011-1	*Streptomyces*	Russia	Trichoptera larvae, lake water	animal
OCH81255 | Gordonia sp. UCD-TK1	*Gordonia*	USA	Ambulatory Surgery Center	clinical sample
PEH75252 | Nocardia sp. FDAARGOS_372	*Nocardia*	USA	Human clinical isolate	clinical sample
OCF84162 | Nocardia brasiliensis	*Nocardia*	Mexico	Human pathogen	clinical sample
OXR45130 | Nocardia cerradoensis	*Nocardia*	Spain	Human knee abscess	clinical sample
AFU00935 | Nocardia brasiliensis ATCC 700358	*Nocardia*	Mexico	Mycetoma of thorax, human lungs	clinical sample
SLL22258 | Mycobacteroides abscessus subsp. Massiliense	*Mycobacteroides*	Australia	Human repiratory system	clinical sample
SLF86799 | Mycobacteroides abscessus subsp. Massiliense	*Mycobacteroides*	Denmark	Human repiratory system	clinical sample
SKY95784 | Mycobacteroides abscessus subsp. Abscessus	*Mycobacteroides*	UK	Human repiratory system	clinical sample
SKN73727 | Mycobacteroides abscessus subsp. Bolletii	*Mycobacteroides*	UK	Human repiratory system	clinical sample
CNG21174 | Mycobacterium tuberculosis	*Mycobacterium*	South Africa	Human sputum	clinical sample
PFX10478 | Nocardia farcinica	*Nocardia*	Spain	Human sputum	clinical sample
PFX05208 | Nocardia farcinica	*Nocardia*	Spain	Human sputum	clinical sample
KRC69836 | Nocardioides sp. Root240	*Nocardioides*	Germany	*Arabidopsis thaliana* leaf	plant
KRC46491 | Nocardioides sp. Root79	*Nocardioides*	Germany	*Arabidopsis thaliana* leaf	plant
KRA92165 | Nocardioides sp. Root682	*Nocardioides*	Germany	*Arabidopsis thaliana* leaf	plant
KRA38205 | Nocardioides sp. Root614	*Nocardioides*	Germany	*Arabidopsis thaliana* leaf	plant
KQZ17413 | Streptomyces sp. Root55	*Streptomyces*	Germany	*Arabidopsis thaliana* leaf	plant
KQY32086 | Nocardia sp. Root136	*Nocardia*	Germany	*Arabidopsis thaliana* leaf	plant
KQX92924 | Streptomyces sp. Root1319	*Streptomyces*	Germany	*Arabidopsis thaliana* leaf	plant
KQX77141 | Streptomyces sp. Root1310	*Streptomyces*	Germany	*Arabidopsis thaliana* leaf	plant
KQP68985 | Microbacterium sp. Leaf288	*Microbacterium*	Switzerland	*Arabidopsis thaliana* leaf	plant
ONH62053 | Frankia sp. CcI49	*Frankia*	Egypt	*Casuarina cunninghamiana* root nodule	plant
KPM56518 | Frankia sp. R43	*Frankia*	USA	*Casuarina equisetifolia* root	plant
ALG08537 | Kibdelosporangium phytohabitans	*Kibdelosporangium*	China	Inner tissue of medical plant *Jatropha curcas* L.	plant
ALG08536 | Kibdelosporangium phytohabitans	*Kibdelosporangium*	China	Inner tissue of medical plant *Jatropha curcas* L.	plant
ALC27020 | Streptomyces sp. CFMR 7	*Streptomyces*	Malaysia	Latex	plant
ONH29850 | Frankia asymbiotica	*Frankia*	USA	*Morella californica* root	plant
PNE14597 | Amycolatopsis sp. BJA-103	*Amycolatopsis*	China	*Valeriana jatamansi* rhizomes	plant
PSK59749 | Streptomyces sp. 111WW2	*Streptomyces*	Australia	Wheat plant root	plant
CQR65882 | Streptomyces leeuwenhoekii	*Streptomyces*	USA	Atacama Desert	soil
OSY35358 | Pseudonocardia autotrophica	*Pseudonocardia*	Tokyo	Atmospheric dust	soil
QBI55540 | Streptomonospora sp. M2	*Streptomonospora*	Germany	Beach sand	soil
ONI75193 | Actinosynnema sp. ALI-1.44	*Actinosynnema*	USA	Chitin enrichment of biocrust soil	soil
KZB79768 | Amycolatopsis regifaucium	*Amycolatopsis*	Australia	Composite arid soil	soil
PWD44862 | Gordonia paraffinivorans	*Gordonia*	Brazil	Compost	soil

CRK51342 l Rhodococcus sp. RD6.2	*Rhodococcus*	portugal	Estuary sendiment	soil
KOX08038 l Micromonospora sp. NRRL B-16802	*Micromonospora*	USA	Field soil	soil
ALV53416 l Streptomyces sp. 4F	*Streptomyces*	China	Garden soil	soil
OUC99979 l Streptosporangium minutum	*Streptosporangium*	South Africa	Garden soil	soil
OXM65930 l Amycolatopsis vastitatis H5	*Amycolatopsis*	Chile	Gravel soil	soil
OXM72510 l Amycolatopsis sp. KNN50.9b	*Amycolatopsis*	Chile	Halite encrusted soil	soil
OUC76591 l Gordonia lacunae	*Gordonia*	South Africa	Lagoon sendiment	soil
KHL16074 l Mumia flava	*Mumia*	Malaysia	Mangrove	soil
KSU58520 l Gordonia sp. SGD-V-85	*Gordonia*	India	Marine sendiment	soil
ASR34495 l Prauserella marina	*Prauserella*	China	Ocean sediment	soil
PBC43470 l Rhodococcus sp. ACPA4	*Rhodococcus*	Norwich	Populus alba organic material	soil
PJN11143 l Streptomyces sp. CB01635	*Streptomyces*	China	Primary forest soil	soil
ARF65131 l Streptomyces violaceoruber	*Streptomyces*	China	Seabed sludge	soil
OPX10399 l Gordonia sp. i37	*Gordonia*	UK	Sediment	soil
EPJ42703 l Streptomyces afghaniensis 772	*Streptomyces*	Afghanistan	Soil	soil
ASO21239 l Actinoalloteichus hoggarensis	*Actinoalloteichus*	Algeria	Soil	soil
ETD33503 l Williamsia sp. D3	*Williamsia*	Antartica	Soil	soil
OZG29444 l Williamsia sp. 1138	*Williamsia*	Australia	Soil	soil
ORM38330 l Williamsia sp. 1135	*Williamsia*	Australia	Soil	soil
ASU58405 l Nocardiopsis dassonvillei	*Nocardiopsis*	China	Soil	soil
ORT56729 l Streptomyces sp. CB03238	*Streptomyces*	China	Soil	soil
ANN18395 l Amycolatopsis orientalis	*Amycolatopsis*	China	Soil	soil
ETA06949 l Gordonia alkanivorans CGMCC 6845	*Gordonia*	China	Soil	soil
EPH43136 l Streptomyces aurantiacus JA 4570	*Streptomyces*	China	Soil	soil
ATE55850 l Actinosynnema pretiosum	*Actinosynnema*	China	Soil	soil
ASR04442 l Gordonia rubripertincta	*Gordonia*	Germany	Soil	soil
AQZ62882 l Nonomuraea sp. ATCC 55076	*Nonomuraea*	India	Soil	soil
OLZ43780 l Amycolatopsis keratiniphila subsp. Nogabecina	*Amycolatopsis*	India	Soil	soil
KSZ58500 l Rhodococcus pyridinivorans KG-16	*Rhodococcus*	India	Soil	soil
QBQ62622 l Saccharomonospora sp. 31sw	*Saccharomonospora*	Iran	Soil	soil
AIG75424 l Amycolatopsis japonica	*Amycolatopsis*	Japan	Soil	soil
OOC01227 l Amycolatopsis azurea DSM 43854	*Amycolatopsis*	Japan	Soil	soil
ONF70734 l Amycolatopsis keratiniphila subsp. keratiniphila	*Amycolatopsis*	Kuwait	Soil	soil
OZV81504 l Micromonospora echinospora	*Micromonospora*	Netherlands	Soil	soil
OLT11829 l Actinomadura sp. CNU-125	*Actinomadura*	Palau	Soil	soil
OXM50056 l Amycolatopsis thailandensis	*Amycolatopsis*	Thailand	Soil	soil
KKD10502 l Streptomyces sp. WM6391	*Streptomyces*	USA	Soil	soil
KOV29961 l Streptomyces sp. XY152	*Streptomyces*	USA	Soil	soil
KOU51254 l Streptomyces sp. MMG1522	*Streptomyces*	USA	Soil	soil
KHD76934 l Actinoplanes utahensis	*Actinoplanes*	USA	Soil	soil
OWY81865 l Rhodococcus sp. BUPNP1	*Rhodococcus*	India	Soil, landfill site	soil
KOY49117 l Gordonia sp. NB4-1Y	*Gordonia*	Canada	Vermicompost	soil
AUA08008 l Streptomyces sp. M56	*Streptomyces*	South Africa	Fungus growing termite nest	soil

TEA08347 | Mycobacteroides salmoniphilum	*Mycobacteroides*	Sweden	Drinking water	water
AJT42459 | Psychromicrobium lacuslunae	*Psychromicrobium*	India	Lake surface water	water
OQR65661 | Streptomyces sp. B9173	*Streptomyces*	Germany	Ocean	water
OHX07254 | Micromonospora sp. WMMB235	*Micromonospora*	USA	Ocean water	water
ODA69298 | Streptomyces sp. AVP053U2	*Streptomyces*	USA	Shallow water marine harbour	water
KHJ70360 | Rhodococcus sp. Chr-9	*Rhodococcus*	China	Tannery wastewater	water
